# Time to Storm the Next CASTLE: Can Atrial Fibrillation Ablation Improve Outcomes in Patients With Heart Failure and Preserved Ejection Fraction?

**DOI:** 10.1007/s11897-026-00786-5

**Published:** 2026-11-05

**Authors:** Alex El Darzi, Maximilian Moersdorf, Michel Abou Khalil, Christian M Massad, Mohammad Montaser Atasi, Yara Menassa, Carlo El Khoury, Nassir F Marrouche

**Affiliations:** 1 Tulane Research Innovation for Arrhythmia Discovery, Tulane University School of Medicine, 1430 Tulane Avenue, New Orleans, LA 70112 USA; 2 Tulane University Heart and Vascular Institute, 1430 Tulane Avenue, New Orleans, LA 70112 USA

**Keywords:** Atrial fibrillation, Catheter ablation, Heart failure with preserved ejection fraction, Atrial fibrosis, Pulmonary vein isolation, Pulsed field ablation

## Abstract

**Purpose of Review:**

Heart failure with preserved ejection fraction and atrial fibrillation frequently coexist and are linked by shared mechanisms including atrial myopathy, elevated filling pressures, fibrosis, and adverse hemodynamic interactions. The combination of both diseases worsens patient outcomes, and catheter ablation has emerged as a potentially important treatment strategy in this population. This review aims to summarize and highlight the current evidence on ablation in patients with atrial fibrillation and HFpEF.

**Recent Findings:**

When examining outcomes of mortality, hospitalization, hemodynamics, and recurrence, ablation appears to be superior to medical treatment with some mixed results. However, no large trials have been conducted on the topic, and no clear guidelines for ablation in HFpEF patients exist.

**Summary:**

Ablation is promising but not yet definitive for patients with atrial fibrillation and HFpEF. Ongoing trials, including CASTLE-HFpEF should clarify the gaps including patient selection, procedural strategy, and outcome benefit.

## Introduction

Heart failure with preserved ejection fraction (HFpEF) is a common disease affecting over 32 million people worldwide, and is associated with decreased quality of life, increased hospitalizations, and mortality [1]. Atrial fibrillation (AF), the most common arrhythmia, affects 59 million people globally and similarly confers increased morbidity and mortality [2]. The two conditions frequently coexist, with AF present in 30–50% of patients with HFpEF in the Framingham Heart Study [3]. The two conditions share a bidirectional pathophysiological relationship, creating a vicious cycle of worsening cardiac function and outcomes [4]. AF impairs left ventricular filling through loss of atrial kick, irregular RR intervals that reduce diastolic filling time, and tachycardia-mediated cardiomyopathy [5]. Conversely, HFpEF promotes AF through elevated left atrial pressures, atrial stretch and dilation, and progressive atrial fibrosis, which create the substrate for arrhythmia perpetuation [6].

Unlike heart failure with reduced ejection fraction (HFrEF), effective treatments for HFpEF remain limited, with only recent trials of SGLT2 inhibitors demonstrating mortality and hospitalization benefits [7]. Several treatment strategies exist for AF, including rhythm and rate control. In patients with HFpEF and AF, treatment with a rhythm control strategy may be particularly relevant because it can break the vicious cycle of HFpEF and AF, potentially providing therapeutic benefit for these patients.

Catheter ablation (CA) is an effective rhythm control strategy for AF, with many studies demonstrating its efficacy in the general and HFrEF populations [8–11]. However, HFpEF is a distinct disease with pathophysiology fundamentally different from HFrEF, so it remains unclear whether these benefits also extend to patients with HFpEF. In this review, we examine the available evidence on CA in patients with HFpEF, focusing on efficacy, the biological rationale, limitations of the current literature, and unresolved procedural questions that are specific to this phenotype.

## Part 1: Understanding the AF-HFpEF Phenotype

### Diagnosis of AF and HFpEF

The coexistence of AF and HFpEF is common, but its true prevalence remains uncertain, with estimates varying widely across studies, from as low as 8% to over 65% [12, 13]. This challenge is partly explained by the marked clinical overlap between AF and HFpEF. Among patients with AF, diagnosing HFpEF is particularly difficult because key diagnostic markers, including echocardiographic parameters and BNP levels, can be influenced by AF itself, limiting their reliability [14]. Various tools including the H2FPEF and HFA-PEFF algorithms have been developed to improve diagnostic accuracy but still carry limitations [15, 16]. Invasive testing in AF patients reveals varying but high levels of indolent HFpEF and is an effective way to screen for HFpEF in AF patients [15]. However, most AF patients do not receive invasive testing, despite it being important for patient outcomes including AF recurrence post-ablation [17, 18]. This underscores the importance of understanding and screening for HFpEF in patients with AF, as it may influence CA strategy and outcomes.

On the other hand, HFpEF patients may have AF (particularly subclinical or paroxysmal AF) that can go undetected without systematic rhythm monitoring. Current guidelines provide no specific recommendations for AF screening in the HFpEF population [19]. This is an important consideration as many of these patients may have undiagnosed AF and may benefit from CA.

### Clinical Impact of AF and HFpEF

The coexistence of AF and HFpEF represents a high-risk clinical phenotype that warrants attention [4]. The Framingham Heart Study showed that patients with AF who subsequently developed HF had markedly increased mortality (HR 2.7 in men and 3.1 in women), while patients with HF who later developed AF also had higher mortality (HR 1.6 in men and 2.7 in women) [3]. A large meta-analysis of nearly 2 million HFpEF patients found that AF independently predicted all-cause mortality (HR 1.21), HF hospitalization (HR 1.32), cardiovascular death (HR 1.38), and stroke (HR 1.87) [20]. Notably, data from the ESC Heart Failure Long-Term Registry revealed that the adverse prognostic impact of AF is particularly pronounced in HFpEF, with AF being associated with a 49% increased risk of HF hospitalization in HFpEF but showed no significant association with worse outcomes in HFrEF [21]. This differential impact was corroborated by a National Inpatient Sample analysis of nearly 5 million hospitalizations, which found AF to be an independent predictor of in-hospital mortality in HFpEF (OR 1.10) [22]. Furthermore, the TOPCAT and PARAGON-HF trials demonstrated that both prevalent and incident AF increase cardiovascular risk in HFpEF [23, 24]. These findings underscore the critical importance of addressing AF in HFpEF patients and provide a strong rationale for investigating rhythm control strategies, including CA, in this vulnerable population.

### Pathophysiology of AF in HFpEF

AF and HFpEF share many common cardiovascular risk factors [25]. These shared risk factors may lead to the development of HFpEF and AF through various mechanisms, including systemic inflammation, microvascular dysfunction, epicardial adipose tissue, hemodynamic changes, and myocardial fibrosis [26]. Systemic inflammation emerges from several of these risk factors through distinct but overlapping pathways. Hypertension, obesity, diabetes, smoking, and alcohol each independently release various pro-inflammatory molecules, thereby sustaining an inflammatory state [27–29]. This systemic inflammation has been directly implicated in AF and HFpEF, with elevated circulating levels of TNF-α, IL-6, and CRP associated with the incidence of both diseases [28, 30].

The exact mechanisms linking systemic inflammation to the development of both diseases remain unclear; however, several mechanisms may explain this link. Microvascular dysfunction and epicardial adiposity, for example, are highly prevalent in both AF and HFpEF and are associated with systemic inflammation [31–34]. Furthermore, circulating inflammatory mediators, such as TGF-β can activate resident cardiac fibroblasts and induce global cardiac fibrosis, which plays a big role in the development of both AF and HFpEF [31]. Atrial fibrosis sustains the reentrant electrical substrate that perpetuates AF, whereas ventricular fibrosis underlies the impaired relaxation and elevated filling pressures that characterize HFpEF [35, 36]. Hence, this concept of global myocardial fibrosis, encompassing both chambers, provides a unifying mechanistic bridge between the two conditions [26].

**Fig. 1:**
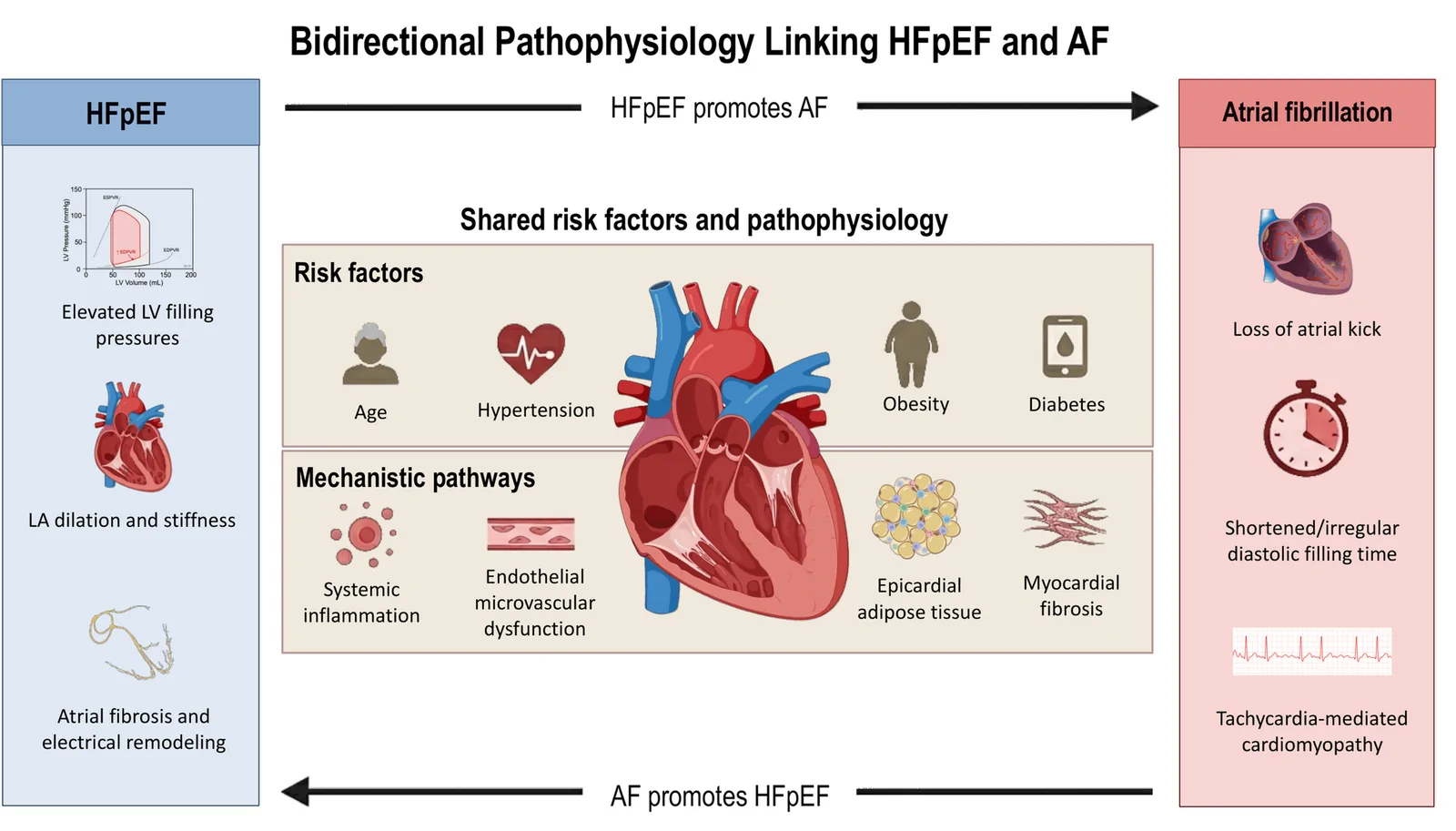
Bidirectional pathophysiology linking HFpEF and AF. Shared risk factors and mechanisms promote both HFpEF and AF. HFpEF promotes AF through elevated filling pressures, LA dilation/stiffness, and atrial fibrosis, while AF worsens HFpEF through loss of atrial kick, irregular filling, and tachycardia-mediated cardiomyopathy. AF, atrial fibrillation; HFpEF, heart failure with preserved ejection fraction; LA, left atrial

Beyond these shared upstream mechanisms, AF and HFpEF can perpetuate one another through a bidirectional vicious cycle of hemodynamic and structural changes [12]. In established HFpEF, elevated left ventricular filling pressures and diastolic dysfunction lead to left atrial dilation and stiffness, promoting atrial fibrosis and ultimately AF [37, 38]. Conversely, AF promotes HFpEF through several mechanisms, including reduced atrial kick and tachycardia-induced cardiomyopathy, which shorten diastolic filling time, elevate left atrial pressure, and worsen diastolic function [39, 40]. To conclude, common mechanisms contribute to the development of both diseases, creating a self-reinforcing cycle in which each condition amplifies the burden of the other (Fig. 1). Understanding the pathophysiology is key to understanding how CA may benefit or harm HFpEF patients.

## Part 2: Clinical Outcomes After Catheter Ablation in HFpEF

### Invasive Hemodynamic Improvements

Invasive hemodynamics is important in patients with HFpEF and AF and serves as a useful marker for assessing treatment outcomes. Chieng et al. randomized 31 HFpEF patients with AF to CA or medical treatment and followed them for 6 months. The patients had an average age of 66 years, with 81% having persistent AF, 29% with a history of hospitalizations, and 19% on diuretics, reflecting patients with a relatively earlier stage of HFpEF [41, 42]. He demonstrated that CA reduced peak exercise pulmonary capillary wedge pressure (PCWP) from 30.4 to 25.4 mmHg (*P* < 0.01), whereas the medical treatment group showed no change (29.5 ± 3.9 to 28.9 ± 5.3 mmHg; *P* = 0.68). Furthermore, improvements in cardiac output and exercise capacity were also observed (peak relative oxygen consumption from 20.2 ± 5.9 to 23.1 ± 7.2 mL/kg/min *P* < 0.01) in the ablation arm [41]. Similarly, Sugumar and colleagues showed that patients who underwent CA had a significant decrease in resting PCWP (12.8 ± 4.3 to 10.0 ± 3.4 mmHg, *P* < 0.05) and peak exercise PCWP (30.3 ± 4.2 vs. 26.8 ± 4.7 mmHg, *P* < 0.01). This effect may be attributed mainly to maintenance of sinus rhythm, with AF free group showing significant improvements in peak exercise PCWP (29.2 to 22.9 mmHg, *P* < 0.01), compared to patients who did not maintain sinus rhythm (31.2 ± 4.5 to 30.0 ± 3.8 mmHg, P = NS) [18].

### Echocardiography and BNP

Echocardiographic studies have yielded mixed but mainly favorable findings regarding structural and functional remodeling following catheter ablation in patients with HFpEF. In patients with persistent AF and HFpEF who maintain sinus rhythm after ablation, significant reverse left atrial morphological remodeling occurs with notable improvements in LA reservoir and contractile strain [43]. Machino-Ohtsuka et al. demonstrated that LV systolic indices, including LVEF and strain, and diastolic indices, such as the E/e’ ratio, improved only in patients who maintained sinus rhythm at follow-up [44]. This effect is mainly observed in patients who revert and stay in sinus rhythm [18, 45]. However, not all studies have shown benefit. A prospective cryoballoon ablation study found that echocardiographic follow-up revealed progression of adverse left atrial remodeling and no meaningful improvement in diastolic function in HFpEF patients, even among those with successful rhythm control [46]. Similarly, natriuretic peptide responses have been heterogeneous, with some studies demonstrating significant reductions in BNP following ablation [41, 47], while others report persistent biomarker elevation even in patients achieving successful rhythm control [46].

### HFpEF Resolution

The concept of “HFpEF resolution” has emerged from multiple studies demonstrating that a substantial proportion of patients no longer meet diagnostic criteria for HFpEF following successful ablation. In one study, 50% of ablation patients no longer met exercise right-heart catheterization criteria for HFpEF, compared with only 7% in the medical therapy arm (*P* = 0.02) [41]. Furthermore, other studies showed HFpEF resolution in 35% of CA patients versus 9% of medically treated patients (*P* = 0.008) [45], and in 45% of patients who underwent CA [18]. These findings suggest that in a subset of patients, AF may be the primary driver of the HFpEF phenotype, and successful rhythm control can effectively “cure” the condition.

### Hospitalization and Mortality

There are no RCTs that directly examine hospitalization and mortality outcomes after ablation in patients with HFpEF, and most of the evidence comes from post hoc analyses of RCTs and observational studies. The CABANA trial analyzed the 778 patients with clinical heart failure, 79% of whom had an EF ≥ 50%. Ablation reduced mortality by 60% (HR 0.40; 95% CI 0.18–0.88), with 4-year mortality rates of 3.3% versus 8.6% for drug therapy [48]. More recently, Martens and colleagues reanalyzed CABANA using the H2FPEF score to identify patients at high risk of HFpEF. The benefit of ablation was observed mainly in patients with high HFpEF probability, where it reduced cardiovascular hospitalization or death by 18% (HR 0.82; 95% CI 0.69–0.98), while no benefit was observed in those with low HFpEF probability [49].

Most observational studies agree with these findings. A Swedish Heart Failure Registry (2005–2019) used propensity score matching and found that catheter ablation was associated with a lower risk of first and recurrent HF hospitalization [50]. In another propensity-matched analysis, 43 HFpEF patients undergoing CA compared to 43 receiving medical therapy alone, the composite of heart failure hospitalization and death was reduced by 70% with CA (HR 0.30; 95% CI 0.13–0.67; *P* = 0.003), driven primarily by a reduction in heart failure hospitalizations [45]. Furthermore, an AF Registry in Japan demonstrated that among 106 propensity-matched pairs of patients with heart failure and EF ≥ 40%, the ablation group had a markedly lower incidence of the composite of cardiovascular death and heart failure hospitalization at 24.6 months (HR 0.32; 95% CI 0.13–0.70; *P* = 0.004) [51]. A large Chinese cohort study by Xie and colleagues similarly demonstrated that ablation significantly reduced rehospitalization for worsening heart failure compared with no ablation among HFpEF patients [52]. A prespecified sub-analysis of the EAST-AFNET4 trial, which included 798 heart failure patients, the majority of whom had HFpEF, demonstrated that early rhythm control therapy initiated within one year of AF diagnosis significantly reduced the composite of cardiovascular death, stroke, and heart failure hospitalization compared with usual care (HR 0.74; 95% CI 0.56–0.97; *P* = 0.03), supporting the broader concept that early rhythm control is beneficial across the spectrum of heart failure subtypes [53, 54].

To place these individual studies in a broader context, several meta-analyses have reported similar outcomes. Bulhões et al. pooled 8 studies including 20,257 patients and found that catheter ablation was associated with lower risks of all-cause death, cardiovascular death, heart failure hospitalization, and all-cause rehospitalization compared with medical therapy in patients with AF and HFpEF [55]. Mahalleh and colleagues similarly found that ablation was associated with a 39% reduction in the composite of all-cause mortality and heart failure hospitalization (HR 0.61; 95% CI 0.43–0.85) [56].

### Stroke

Data on stroke outcomes in HFpEF-specific ablation studies remain limited. The CABANA trial overall showed no significant difference in disabling stroke between ablation and drug therapy arms, but event rates were low, and the trial was not powered to detect differences in thromboembolic endpoints [48]. One observational study reported lower stroke rates in patients with HFpEF who received CA vs. medical therapy (9.63% vs. 16.58%; *p* < 0.01), and similar rates between HFpEF and non-HF patients who received CA (9.63% vs. 8.67%; *p* = 0.65) [57].

### Quality of Life

Quality of life is a primary therapeutic target in AF and HFpEF, where symptomatic burden often defines the patient experience more than any single clinical event. The study by Chieng showed improved quality of life, with the Minnesota Living with Heart Failure score decreasing from 51 to 17 points (*P* < 0.01) [41]. These findings build on earlier observational work by Sugumar and colleagues (STALL AF-HFpEF), which demonstrated that among patients remaining arrhythmia-free after CA, MLHF scores improved by more than 30 points [18]. The CABANA heart failure sub-analysis similarly showed quality-of-life benefits, with the AFEQT summary score averaging 5.0 points higher in the ablation arm over 60-month follow-up (95% CI 2.5–7.4) [48].

### AF Recurrence

Freedom from arrhythmia recurrence is key to the observed clinical benefits. In the CABANA heart failure sub-analysis, ablation reduced AF recurrence by 44% compared with drug therapy (HR 0.56; 95% CI 0.42–0.74) [48]. In the Rattka propensity-matched study, arrhythmia recurrence occurred significantly less often in the ablation group (HR 0.47; 95% CI 0.25–0.87; *P* = 0.016) [45]. A DECAAF II sub-analysis revealed that, after CA, HF, and non-HF patients had the same AF burden at 3 months of follow-up [58]. However, not all studies have shown similarly favorable rhythm outcomes in HFpEF. In a prospective study of 102 patients undergoing cryoballoon ablation, Zylla and colleagues found that HFpEF patients had significantly higher AF recurrence rates than those without heart failure (57% vs. 23%; *P* = 0.003) and a greater need for repeat ablation (39% vs. 14%; *P* = 0.01) [46]. Turagam et al. compared recurrence between patients without HF and those with HFrEF and HFpEF. They demonstrated lower 1-year freedom from atrial arrhythmia in both HFpEF (71.3%) and HFmr/rEF (67.5%) compared with patients without heart failure (79.9%; *P* < 0.001). However, looking specifically at patients with paroxysmal AF, there was no significant difference between the groups (HF: 82.8% vs. HFpEF: 82.4% vs. HFmr/rEF: 71.7%; *p* = 0.09), and in persistent AF, the results were less favorable but still not significant (no HF: 73.3% vs. HFpEF: 64.2% vs. HFmr/rEF: 64.9%; *p* = 0.14) [59]. This suggests that patients with paroxysmal AF, particularly when treated earlier in the disease course, may derive the greatest benefit from catheter ablation, highlighting the potential importance of early intervention. More recently, the AVANT-GUARD trial extended the first-line ablation evidence to patients with persistent AF, showing greater rhythm-control success with pulsed-field ablation than with antiarrhythmic drug therapy [60]. However, whether these findings extend specifically to HFpEF remains uncertain, and dedicated HFpEF-specific data are needed.

### Burden

An even more clinically meaningful outcome than recurrence is AF burden assessment, which has emerged as the new standard for evaluating ablation success [61]. Post-ablation AF burden has been associated with symptoms, hospitalization, and mortality in the general and HFrEF population, with proposed cutoffs ranging from 0.1% to 50% [62–64]. In HFpEF, burden has also been shown to be associated with the incidence of HF hospitalizations, with patients having a burden of less than 0.012% having a higher risk [65]. However, there are no studies for a burden cutoff specific to HFpEF patients post-ablation. Furthermore, for the general and HFrEF population, there is no consensus on a single optimal threshold, underscoring the need for dedicated RCTs to address this gap.

**Fig. 2:**
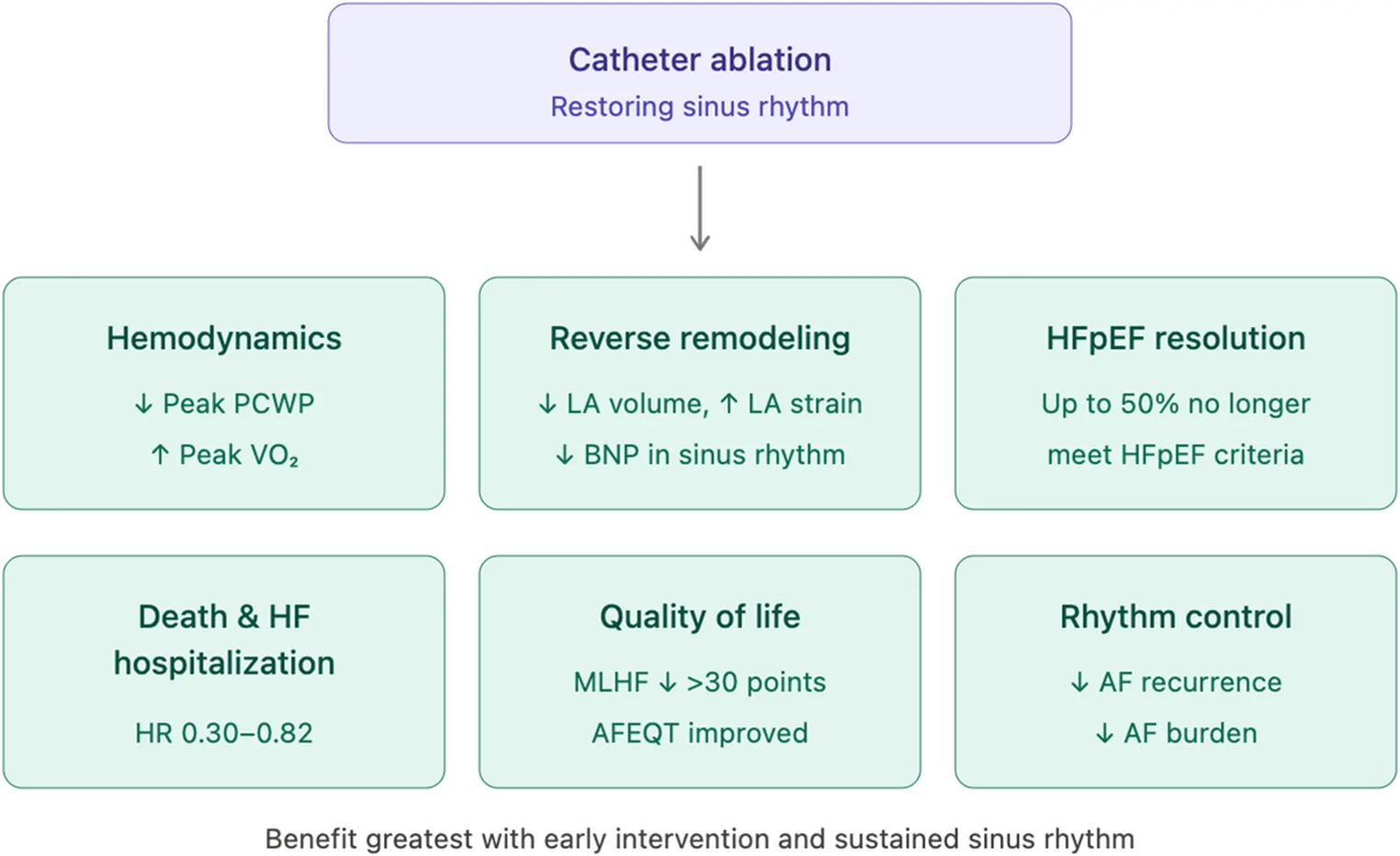
Clinical outcomes of catheter ablation in HFpEF and AF. Catheter ablation may improve hemodynamics, reverse remodeling, HFpEF criteria, hospitalization risk, quality of life, and rhythm control. AF, atrial fibrillation; HFpEF, heart failure with preserved ejection fraction; PCWP, pulmonary capillary wedge pressure; VO₂, oxygen consumption; LA, left atrial; BNP, B-type natriuretic peptide

To conclude, clinical studies of catheter ablation in patients with HFpEF and AF report improvements across multiple outcome domains, including hemodynamics, reverse remodeling, rhythm control, quality of life, and heart failure outcomes, but randomized trials are needed to more definitively assess the role of ablation in this population (Fig. 2).

## Part 3: Special Catheter Ablation considerations

### PVI Only Versus PVI + Additional Ablation Lesions

In the general AF population, randomized trials and large observational cohorts have shown no consistent benefit of extended ablation strategies beyond pulmonary vein isolation, with only a modest improvement in arrhythmia-free survival among patients with persistent AF and significant atrial remodeling [66–70].

Whether these conclusions apply to HFpEF is a separate question entirely, given the pathophysiology of this population. Patients with heart failure have a higher atrial fibrotic burden that extends well beyond the pulmonary veins [71–73]. Notably, several prospective studies have reported higher recurrence rates in HFpEF than in the general population, which may be explained by increased fibrosis and worse hemodynamics that facilitate AF recurrence [74]. This additional substrate may require to adjunctive ablation to reduce recurrence. However, extensive ablation is potentially harmful in HFpEF patients. Extensive ablation risks collateral myocardial injury, impaired residual atrial contractility, and worsening left atrial strain and stiffness [75]. Atrial mechanical function, including left atrial kick, reservoir function, and conduit strain, is of outsized importance in the non-compliant HFpEF ventricle [38, 76], and worsening LA stiffness and strain have been associated with worse CV outcomes, including mortality, in the HFpEF population [77, 78].

Notably, no studies have directly compared PVI alone with PVI plus in HFpEF, leaving this question unanswered. Until prospective data become available, extended ablation beyond PVI should be handled cautiously in this particularly vulnerable population (Table 1).

### PFA Versus RF

Pulsed field ablation (PFA) has emerged as a promising technology that may offer advantages, particularly in patients with HFpEF. Unlike radiofrequency and cryoablation, PFA uses non-thermal energy to create myocardial lesions via irreversible electroporation, potentially resulting in less collateral tissue damage and preservation of left atrial compliance, which is a particularly important function in HFpEF [79, 80]. The MANIFEST-17 K registry, encompassing over 17,000 patients had a major complication rate below 1%, with no esophageal injury, pulmonary vein stenosis, persistent phrenic nerve injury, and a transient phrenic nerve palsy rate of just 0.06% [81]. The ADVENT trial demonstrated non-inferiority of PFA to conventional thermal ablation for both efficacy and safety in paroxysmal AF [80]. A subsequent ADVENT sub-analysis by Reddy and colleagues showed that an AA burden exceeding 0.1% was associated with significantly reduced quality of life and increased clinical interventions, and that more patients achieved an AA burden below this clinically meaningful threshold with PFA than with thermal ablation (OR 1.5; 95% CI 1.0–2.3; *P* = 0.04) [64].

In HFpEF specifically, ablation modality may influence both structural and functional cardiac outcomes. In a comparative study of 223 patients with paroxysmal AF and HFpEF, both RFA and PFA were associated with reductions in left atrial diameter and increases in LVEF, whereas cryoballoon ablation showed no significant structural changes [82]. All three modalities improved quality of life and NYHA functional class, with no significant differences in AF recurrence rates (*P* = 0.964) or procedural complications. Nakatani et al., using serial cardiac MRI in 41 patients undergoing PVI, demonstrated that tissue changes in PFA were more homogeneous with no microvascular damage or intramural hemorrhage, in contrast to thermal ablation, which produced heterogeneous lesions with areas of structural tissue injury. Crucially, the majority of acute LGE had resolved by 3 months after PFA, whereas most LGE persisted after thermal ablation, suggesting a reparative process with less chronic fibrosis following PFA. The LA strain and LA active emptying fraction all declined acutely after both modalities but recovered to baseline only in the PFA group [79]. In the absence of comparative evidence between PFA and RF in patients with HFpEF, preservation of left atrial function remains a compelling theoretical advantage of PFA in this population, which is particularly sensitive to changes in atrial compliance and function.

### What We Learned From the HFrEF Experience

In HFrEF, CA has become an evidence-backed treatment supported by strong randomized data. The landmark CASTLE- AF trial showed that ablation significantly reduced the composite of all- cause death and heart failure hospitalization compared to medication in patients with AF and LVEF ≤ 35%, with all- cause mortality at 13.4% in the ablation group versus 25.0% in the control group, along with notable improvements in LVEF and sinus rhythm maintenance [10]. The CASTLE- HTx trial further extended these findings to the sickest patients awaiting transplant, showing that catheter ablation decreased the combined rate of death, LVAD implantation, and urgent heart transplant in patients with end- stage HFrEF and symptomatic AF [11]. These findings are reflected in guidelines, which provide a Class 1a recommendation for CA in suitable patients with HFrEF [83].

### HFrEF Versus HFpEF

In HFpEF, the underlying pathophysiology differs from that of HFrEF, making it hard to extrapolate HFrEF trial data to this group. Additionally, most evidence comes from retrospective and observational studies, as reflected in current guidelines, which assign CA in HFpEF only a Class 2b recommendation [19]. Nevertheless, several analyses have compared CA outcomes and safety between the two groups. Some retrospective studies found no differences between HFpEF and HFrEF in recurrence, mortality, or hospitalization [84, 85]. Another retrospective study comparing HFpEF and HFrEF ablation observed no significant differences in procedure times (233 vs. 233.5 min), recurrence rates (33.9% vs. 32.6%), adverse events (3.38% vs. 6.62%; *P* = 0.395), improvements in NYHA class (− 0.32 vs- 0.19; *P* = 0.135) and MAFSI symptom severity (− 0.23 vs- 0.09; *P* = 0.116) after ablation [86]. A MANIFEST-PF sub-analysis in HF patients found similar recurrence rates and safety outcomes in the two groups [59]. However, a meta-analysis comparing the two showed that HFpEF did not benefit from CA with respect to all-cause and cardiovascular mortality, whereas HFrEF did [87]. Overall, the data suggest that HFpEF patients may derive the same benefits as HFrEF patients despite their differing pathophysiology, but results are mixed, and further RCTs are necessary.

## Limitations in the Literature and Future Directions

Several limitations exist. First, there are no RCTs assessing HFpEF and CA looking at hard outcomes including mortality, hospitalizations, and stroke. Another major limitation across prior studies is the lack of a uniform definition of HFpEF. Diagnostic criteria varied considerably, ranging from clinical diagnoses to probabilistic HFpEF scoring systems to invasive hemodynamic validation. Furthermore, data on screening and burden of AF in HFpEF, and their link to CA is limited. The field urgently needs large prospective randomized data in HFpEF with hard outcomes. Several trials are currently underway to address this gap, including CASTLE-HFpEF, CABA-HFPEF, and TAP-CHF, each comparing catheter ablation against medical or usual care in patients with AF and HFpEF across a range of clinical and hemodynamic endpoints (Table 1). CASTLE-HFpEF is specifically examining 4000 HFpEF patients and screening them for AF, which is important for understanding the prevalence of undiagnosed AF in HFpEF patients. They will then be randomized into CA vs. medical therapy, and looking at a variety of outcomes, including mortality, strokes, hospitalization, as well as burden and BNP. All patients will receive an ICM post-treatment and be monitored for 3 years, allowing a better understanding of burden in HFpEF patients post-CA. Together, these studies have the potential to do for HFpEF what CASTLE-AF and CASTLE-HTx did for HFrEF. Until their results emerge, the question of whether catheter ablation can meaningfully alter the natural history of concurrent AF and HFpEF has yet to be answered.

**Table 1:** Design, primary endpoints, and evidence gaps addressed by registered trials of catheter ablation for atrial fibrillation in patients with HFpEF

Trial	Design and comparison	Primary endpoint	Evidence gap addressed
CASTLE-HFpEF (NCT07254455)	Screens 4,000 patients with HFpEF/HFmrEF for AF, then randomizes eligible patients to catheter ablation or medical AF treatment.	Hierarchical outcome comprising all-cause death, stroke/TIA, worsening-HF hospitalization, and NT-proBNP reduction.	How prevalent AF is in the screened HF population, and whether ablation improves outcomes versus standard treatment.
CABA-HFpEF (NCT05508256)	Randomizes 1,548 planned patients with AF and symptomatic HFpEF/HFmrEF to first-line ablation or usual medical care.	Cardiovascular death, stroke, and total first and recurrent unplanned cardiovascular hospitalizations for HF or acute coronary syndrome.	Whether ablation reduces recurrent cardiovascular events.
TAP-CHF (NCT04160000)	360 patients planned overall. A 100-patient initial pilot compares ablation with drug-based AF treatment; a subsequent phase compares hemodynamic-guided with empiric HF management.	Time to first HF hospitalization or cardiovascular death within 12 months.	How AF treatment and HF management strategies affect clinical events.

## Conclusions

AF and HFpEF are frequent companions that form a vicious cycle, each amplifying the burden of the other and worsening outcomes. Despite observational studies suggesting improvements in HFpEF after catheter ablation, no randomized controlled trials have been specifically designed to evaluate this population. The ongoing CASTLE-HFpEF, CABA-HFpEF, and TAP-CHF trials are poised to provide the definitive evidence this field urgently needs.

## Key References

- Marrouche NF, Brachmann J, Andresen D, Siebels J, Boersma L, Jordaens L, et al. Catheter Ablation for Atrial Fibrillation with Heart Failure. N Engl J Med. 2018;378(5):417-27.
  - ○ The CASTLE-AF trial established catheter ablation as an outcome-modifying therapy in patients with AF and HFrEF, providing the key comparator framework for asking whether a similar “CASTLE” effect may exist in HFpEF.
- Chieng D, Sugumar H, Segan L, Tan C, Vizi D, Nanayakkara S, et al. Atrial Fibrillation Ablation for Heart Failure With Preserved Ejection Fraction: A Randomized Controlled Trial. JACC Heart Fail. 2023;11(6):646-58.
  - ○ This randomized trial provides the strongest direct evidence in AF-HFpEF, showing that catheter ablation can improve invasive exercise hemodynamics, symptoms, quality of life, and HFpEF diagnostic status compared with medical therapy.
- Sugumar H, Nanayakkara S, Vizi D, Wright L, Chieng D, Leet A, et al. A prospective STudy using invAsive haemodynamic measurements foLLowing catheter ablation for AF and early HFpEF: STALL AF-HFpEF. Eur J Heart Fail. 2021;23(5):785-96.
  - ○ This prospective study links successful rhythm control after ablation with improvements in resting and exercise pulmonary capillary wedge pressure, supporting a mechanistic benefit of ablation in early HFpEF.
- Packer DL, Piccini JP, Monahan KH, Al-Khalidi HR, Silverstein AP, Noseworthy PA, et al. Ablation Versus Drug Therapy for Atrial Fibrillation in Heart Failure: Results From the CABANA Trial. Circulation. 2021;143(14):1377-90.
  - ○ This CABANA heart failure sub-analysis provides important post-hoc evidence that ablation improves survival, AF recurrence, and quality of life in patients with AF and heart failure, most of whom had preserved ejection fraction.
- Rillig A, Magnussen C, Ozga AK, Suling A, Brandes A, Breithardt G, et al. Early Rhythm Control Therapy in Patients With Atrial Fibrillation and Heart Failure. Circulation. 2021;144(11):845-58.
  - ○ This EAST-AFNET 4 heart failure sub-analysis supports the broader principle that early rhythm control reduces adverse cardiovascular outcomes in patients with AF and heart failure, including many patients with preserved ejection fraction.

## Data Availability

No datasets were generated or analysed during the current study.
